# Digital Pandemic Stress in Higher Education in Venezuela

**DOI:** 10.3390/ejihpe12120132

**Published:** 2022-12-13

**Authors:** Álvaro Antón-Sancho, Diego Vergara, Elsy Medina, María Sánchez-Calvo

**Affiliations:** 1 Technology, Instruction and Design in Engineering and Education Research Group, Catholic University of Avila, C/Canteros s/n, 05005 Avila, Spain; 2 Facultad de Ciencias de la Educación, Campus Barbula, Universidad de Carabobo, Valencia 2001, Venezuela

**Keywords:** anxiety, digital resources, digital learning environments, Global Innovation Index, Latin America, digital stress

## Abstract

The COVID-19 pandemic had a great impact on the process of integrating digital technologies in higher education and caused digital stress among professors, mainly in countries with a lower level of digitalization. In this work, quantitative research was carried out on the stress of professors in Venezuela due to the digitalization of their teaching activities caused by the pandemic, and gender gaps were identified in this regard. This digital stress was compared with that of professors in other countries with a low level of digitalization. For this purpose, a questionnaire designed by the authors was used. The questionnaire was distributed to a sample of 129 Venezuelan professors and 132 professors from countries with low digitalization levels. As a result, it was found that Venezuelan professors have lower digital competence and lower digital stress than their colleagues in weakly digitized countries, and that digital stress decreases as digital competence increases. Moreover, among Venezuelan professors, there was a strong gender gap in digital stress, which was higher among females in all subject areas, except for Health Sciences. This gender gap is specific to Venezuela since it differs from that in countries with low digital levels. According to the results, we urgently recommend investing resources in the digital training of faculty members, especially in regards to the integration of female professors.

## 1. Introduction

### 1.1. Context and Approach

In March 2020, the World Health Organization identified the COVID-19 pandemic to be a global health crisis [1]. The virus spread rapidly and forced governments in much of the world to control it, taking extraordinary measures, which conditioned logistical, cultural, and economic decisions [2,3]. The educational sphere was no stranger to this reality and efforts to mitigate the spread of the virus among the youth and adult populations caused 150 countries to close their educational institutions by the end of March 2020, affecting 80% of the world’s student population [4]. Children and university students experienced an unprecedented disruption in their educational experiences, having to adapt very quickly to home-based education, online learning, and/or other new ways of instruction [5]. This meant that about 94% of the student population and millions of professors around the world adapted to online learning in an immediate and unexpected way in order to continue learning and teaching [6].

As far as professors are concerned, teaching has often been considered one of the most stressful jobs in the professional landscape, and the responses that professors have been forced to offer in the wake of the pandemic have added to the list of new stressors that professors must face, including those arising from the emergence of online teaching [7]. At a time when digital tools provided the only access to education, both infrastructure and technical skills were in short supply [8]. The rapid shift to virtual learning implied the need for faculty to communicate digitally; integrate technological tools; design online instructions; assess students’ comprehension levels; serve them equitably from the virtual environment; design and develop didactic materials; and implement tasks and provide learning experiences to students to enable their interaction with each other, the professor, the content, and the technology in a synchronous and asynchronous way [9]. 

This digital transformation has affected all levels of education, including higher education [10]. The digitization of training processes in higher education has been carried out in different ways and with different intensities depending on the geographical area involved [11,12]. In this sense, the main setbacks generated by the intensification of the use of these online educational tools have been in facing different challenges, such as accessibility, flexibility, learning pedagogy, and educational policy. In addition, many countries showed substantial problems with having an adequate Internet connection and ensuring their students can access digital devices, as was the case of developing countries [13]. The already existing digital divide was widened according to one’s access to multiple mobile devices or the availability of fast Internet [5]; thus, the pandemic has had a great impact on the world’s education system, particularly in geographic areas with a low level of digitization and innovation, which have a lower capacity to provide distance education and training to professors [14]. Likewise, the specialized literature has identified a gender gap that puts females at a disadvantage as one of the main dimensions of the digital divide, which unequally affects different geographical regions according to the cultural stereotypes that affect each country [15]. 

The different described dimensions of this digital divide have caused socio-emotional problems, anxiety, and stress derived from the abrupt need to use sophisticated digital technologies due to the pandemic. These problems include areas of the affective dimension of the agents involved in higher education, such as resilience [16], insecurity [17], lack of control and anxiety [18], irritability [19], difficulties in establishing online communications [20], nervousness [21], and fear [10]. These types of problems, hereafter referred to as “digital stress”, have affected both higher education students [20,22] and professors [12,23,24]. 

The level of innovation of the different economies, which is strongly correlated with the level of their digitization, has been used as a discriminating criterion for the impact of the pandemic on the digitization process in different countries and the digital stress it has caused among professors [12,25]. A universal way of measuring this level is through the Global Innovation Index (GII) [26], developed by Cornell University, the Institut Européen d’Administration des Affaires (INSEAD, Fontainebleau, France), and the World Intellectual Property Organization (WIPO, Geneva, Switzerland), which analyzes the innovative and digital character of different countries of the world, distributed in seven geographical areas. Thus, the area of Latin America and the Caribbean, which is the focus of this study, corresponds to GIIs that range between 22.8 and 36.1 on a scale of 0 to 100. In this Index, Nicaragua, Suriname, and French Guiana are left out, while Venezuela left the list in 2016, the year in which Venezuela’s GII was 22.3, the lowest in the region. For that reason, the works that have studied the impact of the pandemic on the digitization process in Latin American and the Caribbean taking into consideration different GIIs have not incorporated these countries in the scope of their studies [12,24]. 

This paper analyzes the level of digital stress caused by the COVID-19 pandemic among university professors in Venezuela and identifies gender differences accordingly. In addition, a comparative study is made of the digital stress of Venezuelan professors with that expressed by university professors in counties with low GIIs in Latin America and the Caribbean. For this purpose, a questionnaire designed by the authors was used, and the responses to which were analyzed quantitatively.

### 1.2. Digital Transition in Latin America and the Caribbean

Several international organizations have stated that digital transformation in Latin America and the Caribbean must be considered in the long term, since technological changes there are developing more slowly than in other regions of the world. This is evidenced by data such as the following [27]: (i) 68% of the total population of Latin America had access to the Internet in 2018, well below the average of OECD countries, which reached 84% in terms of access that same year; (ii) 75% of the richest population in Latin America uses the Internet and only 37% of the most disadvantaged population uses it, with this difference between the rich and poor being much greater (almost 40 percentage points) than that of OECD countries. This led to difficulties in advancing digitization during the development of the pandemic. In addition to this digital divide, professors in Latin America and the Caribbean have had to face other problems linked to the geographical characteristics of their region, as it is very extensive in terms of territory and population, with a high dispersion of the population and unequal access to technological resources [28]. This has resulted in difficulties in providing education during the pandemic due to the generalized socioeconomic differences among students, caused by unequal access to technology, connectivity, and digital resources [29]. Finally, the digital competencies developed by professors, conditioned by their experience in the use of ICT, have been shown to be insufficient or improvable [30], and it was also observed that inequalities in the use of digital skills by professors were conditioned by gender or age [31]. The aforementioned factors constitute evident limitations in the digital development of Latin America and the Caribbean, affecting the integration of ICT into the teaching profession [32] and conditioning the way in which professors developed their work during the pandemic.

The GII analyzes the level of innovation and digitization of the economies of 131 countries throughout the world and assigns each of them an index between 0 and 100 [26]. Thus, the different countries are classified into seven zones, which are defined by geographic location so that the GII is roughly homogeneous in each of them. One of these seven zones is Latin America and the Caribbean. In 2021, 15 economies were analyzed in this zone, with GIIs as shown in Table 1. 

**Figure 1:**
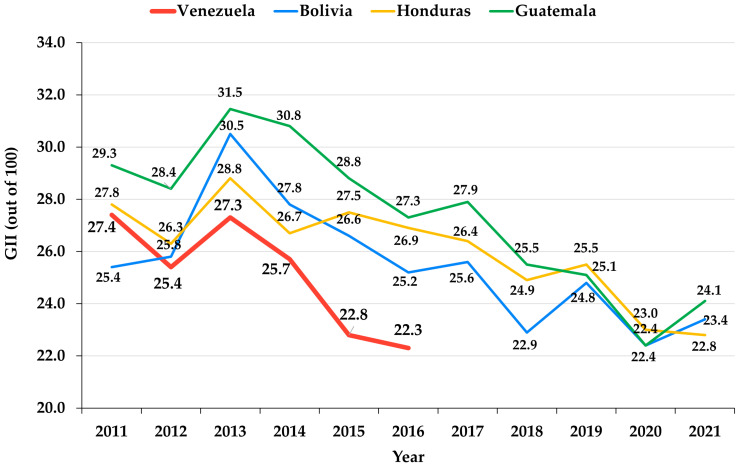
Evolution of the GII of Venezuela and countries with a low level of digitization, Guatemala, Bolivia, and Honduras, in the period 2011–2022.

The mean GII in Latin America and the Caribbean in 2021 was 29.29, with a standard deviation of 4.55. If one defines a low GII as that which is lower than the mean GII minus the standard deviation [12]—i.e., lower than 24.74, according to the 2021 index—, it follows from the data in Table 1 that the countries with low GIIs are Guatemala, Bolivia, and Honduras. On the other hand, the last year when Venezuela was evaluated by the GII was 2016, being at that time among the countries with the lowest GIIs in the region and having suffered a gradual decline in its indices in previous years (Figure 1). The evolution of the digitization process of economies with low GIIs in the last 10 years (Figure 1) has been reasonably homogeneous among them [33]. Since 2013, when they experienced a sharp increase, there has been a downward trend in all of them with very similar slopes. In the case of Venezuela, both its indices and the trend in their variation have been behaving similarly to those of countries with low GIIs, although with lower indices (Figure 1). However, the absence of data on Venezuela’s GII as of 2016 prevents us from concluding that the process of digitization integration in this country and the impact of the pandemic in this regard has occurred in a manner analogous to that of countries with low GIIs.

In the specific case of Venezuela, the experiences of the digitization of higher education collected in the literature reveal the existence of certain limitations for the integration of ICT into teaching, such as the following [34]: (i) the scarcity of options available to professors in terms of the possibility of using communicative tools or collaborative resources as well as the technical difficulties in establishing streaming communications; (ii) difficulty accessing digital technologies by professors and students; (iii) technical inability to create repositories of educational materials with Creative Commons licenses; and (iv) limited resources of universities to provide adequate training in digital competence to their professors. All of these are caused by limitations in access to basic services, including the Internet and electricity, the lack of investment in infrastructure, and the country’s hyperinflationary situation [35]. In turn, these circumstances explain why there has not been, in the case of Venezuela, such an agile response to the process of the digitization of higher education as in other, more digitized countries in the same region [36]. Data from the Inter-American Development Bank suggest that the situation in Venezuela is not likely to improve in the coming years, due to certain factors that specifically affect Venezuela and strongly contract its economy [37,38]: (i) a stable hyperinflation; (ii) the high degree of state interventionism, which has a negative impact on legal security and incentives to invest in the country; (iii) strong international sanctions, which strangle Venezuela’s economy; and (iv) other factors affecting the country’s economy, such as the collapse of the oil sector. 

Some authors believe that, in fact, the digitization of teaching and learning processes in higher education in Latin America and the Caribbean has typically been marked by a gender gap that puts female professors at a disadvantage [39]. This gap stems from cultural stereotypes strongly established in the societies of the region, according to which there is a precise definition of gender roles [40]. Since the technological and digital domain is classically attributed to men, this gender gap penalizes women in terms of access to technologies and training in the use of these technologies, both before and after the pandemic [41,42,43]. As a consequence of this gender gap, the literature reveals the existence of significant gender differences in the digital competence levels of professors, with lower levels among women [44]. In the specific case of Venezuela, this gender gap, together with other forms of social inequality of a cultural or economic nature, has increased since the COVID-19 pandemic, which has led to an intensification of inequalities in the use of digital technologies among professors [45].

### 1.3. Digital Stress in Higher Education

First, it is necessary to mention that the literature distinguishes between two types of stress: distress and eustress [46]. The term eustress refers to a feeling of healthy tension that prompts action to overcome an obstacle or difficulty. It is, therefore, a controlled, healthy stress and helps to carry out a correct activity. Instead, distress consists of negative stress, which involves aspects of an affective dimension, such as nervousness, anxiety, or a feeling of being incapacitated. From the physical and emotional point of view, it is a negative sensation, and, from the point of view of action, it does not help or motivate, but leads to feeling discouraged and blocked. When there are high levels of prolonged distress caused by an imbalance between one’s own skills and the requirements of the job, this distress can give rise to so-called burnout, which is a multidimensional syndrome, usually included among psychological syndromes, characterized by extreme emotional exhaustion, depersonalization (development of negativity and insensitivity), and reduced personal accomplishment, which may be related to episodes of depression [47]. From now on, the word stress will be used in this manuscript to refer to this type of negative stress: distress.

The set of demands mentioned above generated unprecedented stress, constituting a threat to the short- and long-term well-being of professors, many of whom were already facing stress in their personal lives [48]. Although this term has been one of the most controversial constructs in the field of psychology, it is now perfectly defined by the scientific community, considering it as a chronic stress response consisting of three essential factors: emotional exhaustion, depersonalization, and low personal fulfillment [49]. This type of stress can lead to a series of cardiovascular alterations and psychoneuroimmunological changes and can be accompanied by anxiety-related behaviors, sleep disorders, and a low general perception of one’s health status [50]. There is a general consensus in empirical research conducted in several countries around the world, such as the United Kingdom, USA, Brazil, Mexico, Australia, Spain, and Portugal, which shows that professors, as a result of the pandemic, have suffered significantly in terms of their psychological state, as well as their physical and professional well-being [48,51,52], reducing their perception of well-being in the profession and generating concern about their professional future [53]. This type of situation deserves special consideration, as prolonged experiences of stress can lead to burnout, which in turn is associated with a reduction in professional confidence and even quitting [54,55].

The effects of prolonged stress derived from the digitization process on professors have been widely studied; several investigations have exposed that stress and depressive symptoms in professors negatively influence the socio-emotional and language development of their students, reduce their commitment to supporting the facilitation of student learning [56], limit their positive beliefs about their students and teaching practice [57]. Additionally, they adopt leadership that prevents autonomy in learners [58], develop negative perceptions about their ability to control their students’ behavior and manage the classroom effectively, which has been interpreted as a feeling of depersonalization, feel a greater disregard for teaching, and feel a sense of loss in their achievements as a teacher [59,60].

Accordingly, numerous studies have shown that stress is positively related to emotional exhaustion and negatively related to personal fulfillment, so that training in emotional intelligence can be considered a relevant factor in the prevention of burnout syndrome, helping to ensure the mental well-being of professors [61,62,63]. Therefore, it is possible to reduce professors’ level of emotional exhaustion and increase their productivity [64,65,66], as well as increase their job satisfaction and involvement [66,67,68], through specific training focused on improving their emotional competence [69]. Emotional exhaustion is responsible for a decrease in cognitive performance and health degradation, which must be addressed [70], even more so if we consider the benefits of overcoming a state of burnout and improving the well-being of professors who are the determining factors of educational quality in a country. Therefore, if one conceives emotional intelligence as a capacity of individuals to regulate their emotions, strengthening professors’ decision-making in teaching situations would improve their teaching practice and, therefore, pave the way for their success in the educational field [71].

Regarding the integration of digital tools into the teaching processes in higher education and the consequent stress derived from it, studies from the literature have found that the area of a professor’s expert knowledge is a strongly explanatory variable. In this sense, the area of knowledge partially explains the levels of digital skills manifested by the professors [72,73], as well as the patterns of digital technology usage on their part [74]; it is more frequently used among professors of technical areas, who present higher levels of digital competence. However, a frequently indicated source of digital stress comes from the deficiency in teachers’ techno-pedagogical skills, for which their area of knowledge is not an explanatory variable [75,76,77].

In short, due to the COVID-19 pandemic, an unplanned pedagogical change was urgently required and this manifested in the adoption of online teaching, which involved the forced use of technology by the teaching staff, who became crucial actors in providing a successful educational response to the pandemic [59]. This provided an opportunity to rethink education, not just to improve schooling, but to focus on what, where, and how to learn [78]. The answer to the how of this question has been taken up by professors, who have been forced to change their practices from traditional forms of teaching to online instruction, which has required the use of emotional strategies to manage the anxieties experienced when using online tools [79]. This answer has to do not only with the educational system in question, but also with the process of the integration of technological tools in the region—in this case, in Latin America [80]—and with broad and deep aspects of the social structure, such as gender stereotypes or the differences between public and private ownership of universities [81]. The pandemic and the level of digital development in each region, focusing on the study of Latin America and the Caribbean, have reduced professors’ perceptions of well-being in their profession [12]. The stress generated in this profession has led us to reflect on the need to develop interventions to improve their mental comfort, facilitating emotional management and the development of digital skills, which requires a strong investment in policies for the reform and renewal of the teaching profession [53] to ensure the high quality of the education system.

### 1.4. Objectives and Variables

The general objective of this work is to study the incidence of stress generated by the COVID-19 pandemic related to the use of digital teaching technologies among university professors in Venezuela, to identify possible gender gaps in this digital stress, and to compare it with that of professors in Latin American countries with low levels of digitization. Specifically, the following objectives are sought: (i) to assess the level of digital stress perceived by university professors in Venezuela due to the pandemic and to identify the dependency relationship that these levels may have on the digital competence of professors and their assessment of the professional teaching aspects linked to digitization; (ii) to compare the digital stress of Venezuelan professors with that of their colleagues in countries with low digitization indices; and (iii) to identify gender gaps in the levels of digital competence and stress derived from the pandemic among Venezuelan professors and to compare these gaps with those existing in countries with low GIIs.

**Figure 2:**
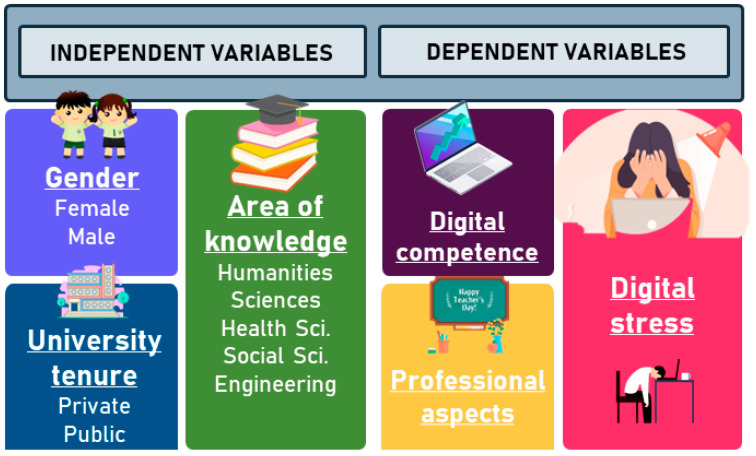
Research variables of the study.

For this purpose, three independent variables, all of which are nominal in nature, are defined as follows (Figure 2): (i) the main independent variable is gender and is dichotomous, with the values being either female or male; and the secondary independent variables are: (ii) area of knowledge and (iii) university tenure. The area of knowledge is a polytomous variable whose values have been extracted from the International Standard Classification of Education of the UNESCO Institute of Statistics [82], but integrating Education within the area of Social Sciences. Specifically, for the purposes of this paper, the following areas are distinguished: (i) Arts and Humanities (specifically, philology, literature, philosophy, art, and history; hereafter, Humanities); (ii) Sciences (specifically, mathematics, natural and experimental sciences); Health Sciences (specifically, medicine, nursing, and veterinary; hereafter, Health); (iii) Social and Legal Sciences (specifically, economics, law, political science, geography, communication, sociology, education, pedagogy, and psychology; hereafter, Social Sciences); and (iv) Engineering and Architecture (which covers technical education; hereafter, Engineering). Finally, university tenure is a dichotomous variable whose possible values are private or public.

Three dependent variables will be studied in this work: (i) the self-perceived level of digital competence of university professors; (ii) an assessment of professional aspects linked to the process of integrating digital technologies in higher education classrooms during the pandemic (support provided by the university, with technical equipment available and training received); and (iii) the digital stress of professors due to the increased use of digital technologies due to the pandemic. The three variables are ordinal quantitative and are measured on a one to five Likert scale, in which one means the lowest rating and five means the highest.

## 2. Materials and Methods

### 2.1. Participants

The sample was obtained by a non-probabilistic convenience sampling process among university professors in Venezuela and in Latin American and Caribbean countries with low GIIs. The only inclusion criterion was to have been a practicing professor at a university in Venezuela, Bolivia, Honduras, or Guatemala at least since before the global declaration of the COVID-19 pandemic in 2020. A total of 276 professors were contacted by the authors, of whom 261 (129 from Venezuela and 132 from countries with low GIIs) responded to the questionnaire that was sent to them as a research instrument. All participants gave their answers voluntarily, freely, and anonymously. All obtained responses were validated.

**Figure 3:**
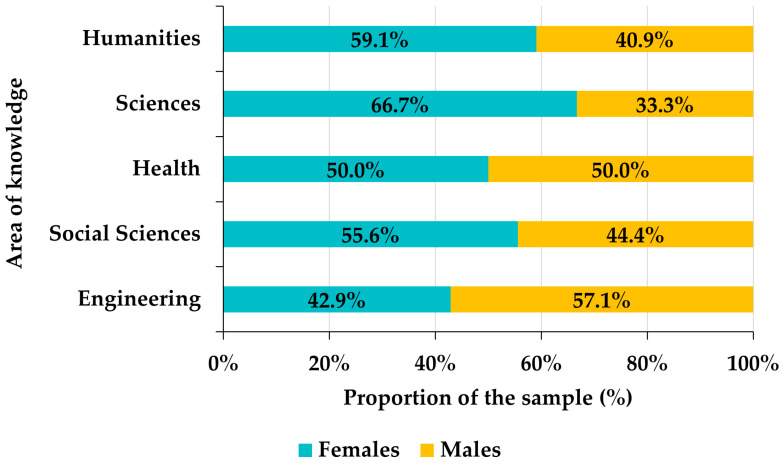
Distribution of Venezuelan participants by areas of knowledge.

**Figure 4:**
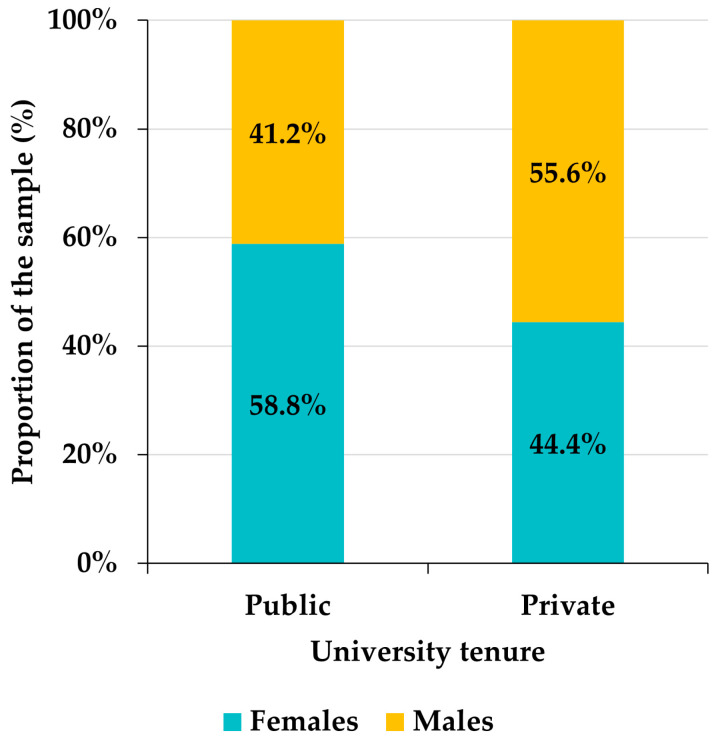
Distribution of Venezuelan participants by university tenure.

Among the 129 Venezuelan participants, 55.8% were female and 44.2% were male. Although there is a slight superiority in the frequency of females with respect to males, the Pearson goodness-of-fit test allows us to assume that the distribution of the sample by gender is approximately homogeneous (chi-square = 1.7442, df = 1, *p*-value = 0.1866). However, there is a certain gender bias in the distribution of participants by areas of knowledge among the Venezuelan faculty (Figure 3). Indeed, in the areas of Humanities, Sciences, and Social Sciences, there is a notable superiority of females (10 percentage points in the case of Social Sciences, almost 20 in Humanities and around 30 in Sciences), the gender distribution is uniform in Health Sciences, and in Engineering the proportion of males exceeds that of females by about 15 percentage points (Figure 3). This bias is statistically significant (chi-square = 24.527, df = 4, *p*-value < 0.0001). Likewise, the frequency of males exceeds that of females by slightly more than 10 percentage points in private universities, while, in public universities, females outnumber males by more than 15 points (Figure 4). Again, gender and university tenure biases are statistically significant (chi-square = 19.688, df = 1, *p*-value < 0.0001).

**Figure 5:**
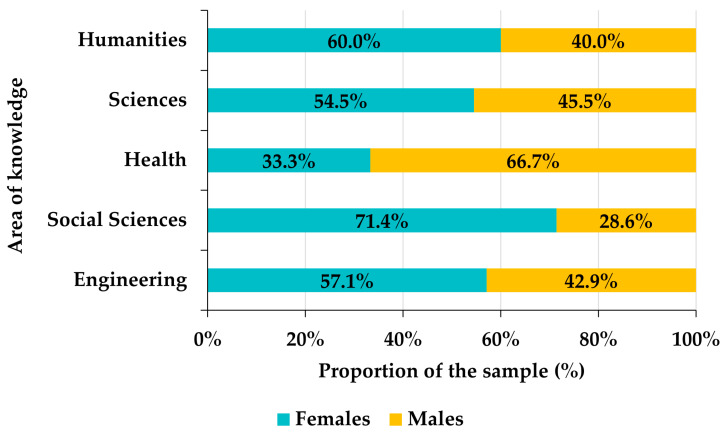
Distribution of participants from countries with low GIIs—Bolivia, Honduras, and Guatemala—by areas of knowledge.

**Figure 6:**
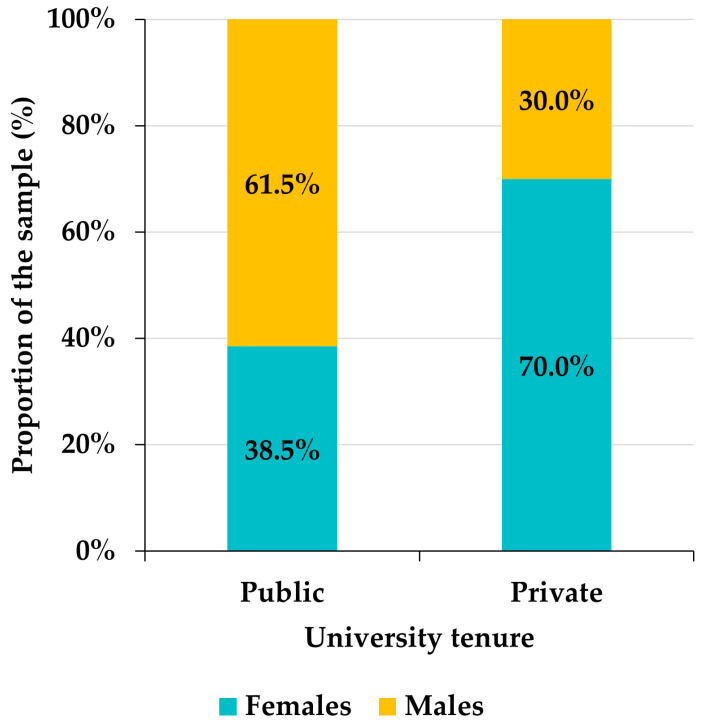
Distribution of participants from countries with low GIIs—Bolivia, Honduras, and Guatemala—by university tenure.

There are 132 participants from low-GII countries (77 from Bolivia, 31 from Honduras, and 24 from Guatemala), distributed as 57.6% females and 42.4% males, which represents an approximately homogeneous distribution by gender (chi-square = 3.0303; df = 1; *p*-value = 0.0817). In these countries, the male professors are more represented in Health Sciences, while in the rest of the areas, females are more represented (Figure 5). Finally, female professors are in the majority in private universities, with a proportion more than double that of male professors, while in public universities, female professors slightly exceed half the percentage of male professors (Figure 6).

### 2.2. Instrument

A questionnaire designed by the authors for the purposes of this research (Table 2) was distributed to the two samples of participants. 

The questionnaire consists of 22 questions differentiated into three different scales, which correspond to the three dependent variables analyzed (Table 2): (i) questions about professors’ digital competence (items 1 to 11) which entails an overall assessment of their digital skills, ability to adapt to digital learning environments, capacity for continuous learning, digital communication skills, creativity in the use of digital learning environments, knowledge of information management, network leadership, ability to guide didactically the use of digital learning resources, resilience, teamwork skills, and strategic vision; (ii) questions asking professors to assess the different professional aspects that influence their adaptation to virtual learning environments during the pandemic (items 12 to 14), including support from the university in the digitization process, technical equipment of the university, and training received in the digitization of teaching activities; and lastly, (iii) questions to assess the level of teaching stress derived from the need to adapt to digital learning environments as a result of the pandemic (questions 15 to 22), including insecurity, anxiety, feeling that difficulties are growing, feeling of inability to face the new challenges of the digitalization of teaching activities, irritability, nervousness, feeling the quality of work is influenced by anxiety from the risk of contagion, and feeling of not being in control of the situation. All responses were measured on a quantitative Likert scale from 1 to 5, in which: 1—null; 2—low; 3—moderate; 4—high; and 5—very high. 

### 2.3. Procedure and Data Analyses

**Figure 7:**
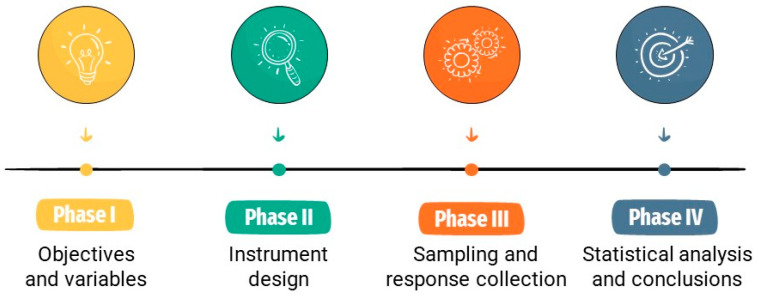
Research phases.

This work is a quantitative research study based on the responses of a sample of 129 university professors from Venezuela and 132 professors from Latin American countries with low GIIs to a questionnaire which was designed as an instrument. The same questionnaire, described in the previous section, was administered to the two samples of participants. This questionnaire consisted of three groups of questions, which serve to quantitatively measure on a Likert scale of 1 to 5 the evaluations of the participants from each of the regions analyzed in relation to the three variables that are the focus of this research: (i) digital competence; (ii) professional aspects linked to the development of digital stress; and (iii) levels of digital teaching stress. The satisfactory achievement of the research objectives was therefore achieved through statistical analysis of the responses and, in particular, by comparing the results of the two samples analyzed. The following phases were followed (Figure 7): (i) determination of the objectives and definition of the research variables; (ii) design of the questionnaire; (iii) sampling and collection of responses; and (iv) statistical analysis of the data and drawing of conclusions. 

Data collection was carried out in accordance with the Declaration of Helsinki. The participants were informed of the research objectives of the questionnaire prior to their participation, for which they gave their express consent. Participation was voluntary, free, and anonymous, and no personal data were collected that could lead to the identification of the participants, something of which the participants were also previously informed. The validation of the instrument was carried out in two phases: (i) factor analysis, to determine the latent factors that explain the responses and the variance explained by the model; and (ii) determination of the level of internal consistency and reliability of the model resulting from the factor analysis, by means of the Cronbach alpha and composite reliability parameters. An exploratory factor analysis (EFA) was carried out with the responses to identify the latent factors that could explain the responses to the questionnaire. The factors detected by the EFA were confirmed by a confirmatory factor analysis (CFA) statistics. In addition, the reliability of the questionnaire was analyzed using Cronbach’s alpha and composite reliability (CR) parameters. For the analysis of the responses, the main descriptive statistics were computed, and it was verified, by means of the Lilliefors normality test, that the responses were not normally distributed. For this reason, the nonparametric bilateral Wilcoxon test for comparison of means was chosen to compare the responses of professors from Venezuela and those from countries with low GIIs and to identify gender gaps in the mean responses of the different groups of questions of professors from both regions. A linear regression model was used to compare the results obtained in the Venezuelan sample and in the sample of countries with low GIIs and to analyze the degree of dependence of the levels of digital stress on the rest of the dependent variables considered. Finally, to identify gender gaps in the responses given within the different areas of knowledge and within the different university tenures of the Venezuelan professors and those from countries with low GIIs, a multifactor ANOVA test was used. All hypothesis contrast tests were carried out with a significance level of 0.05.

## 3. Results

### 3.1. Factor Analysis

The results of the EFA confirm that there are three latent factors that explain the instrument used in the research (Table 3). 

These three factors determine the three scales of questions that have already been defined: (i) digital competence (factor 1); (ii) assessment of professional aspects related to the process of digitalization of teaching activities (factor 2); (iii) digital stress due to the pandemic (factor 3). This distribution of the questions into three scales explains 63.9% of the total variance (Table 4). The responses obtained from the questionnaire have internal reliability, given that the Cronbach’s alpha parameters and the CR coefficients are all above 0.7 (Table 5).

### 3.2. Analysis of Responses

#### 3.2.1. Descriptive Results for the Venezuelan Case and Comparison between Venezuela and Low-GII Countries

In general, the Venezuelan professors surveyed gave an intermediate–high rating to their digital competence, with the smallest deviation among the three scales, the smallest coefficient of variation, and a slight asymmetry to the right (Table 6). Their assessment of the professional aspects linked to the digitization of their teaching activities stood out for three reasons: (i) it is lower than that of their digital competence; (ii) it shows less variation; and (iii) it is distributed approximately symmetrically. Finally, the Venezuelan professors estimated that their level of stress regarding teaching digitalization is intermediate–low, although on this scale the variation is the greatest of all, exceeding 50%, and the distribution of the responses shows a certain asymmetry to the left (Table 6). The Lilliefors normality test statistics (Table 7) confirm that none of the groups of responses are normally distributed.

The mixed linear regression model showed that there is a significant linear dependence between stress, digital competence, and the evaluation of the professional aspects required in the digitalization of teaching activities. Specifically, digital stress increases the lower the digital competence expressed and increases slightly the higher the valuation of professional aspects, with the slopes indicated in Table 8. 

This linear regression model explains in a statistically significant way the relationship between the three groups of responses (residual standard error = 1.29 on 383 degrees of freedom; multiple R-squared = 0.0724; *F*-statistic = 14.99; *p*-value < 0.0001). In contrast, the pandemic-derived digital stress of professors from low-GII countries cannot be significantly explained solely on the basis of their digital competence and a valuation of the professional aspects related to digitization (Table 9), but there must be other sociodemographic or academic factors that are influential, given that the *p*-value of the regression model is greater than the significance level (residual standard error = 1. 16 on 393 degrees of freedom; multiple R-squared = 0.0136; *F*-statistic = 2.71; *p*-value = 0.0680).

The Venezuelan professors reported having a digital competence 9.14% below the average digital competence of their colleagues in countries with low GIIs and rated the professional aspects linked to digitalization 18.28% lower than them (Table 10). 

Moreover, with the levels of digital stress expressed being intermediate–low in both cases (the ratings are slightly below 2.5 out of 5), the Venezuelan professors expressed a digital stress that is 10.89% lower, on average, than that expressed by professors in countries with low GIIs (Table 10). From the Wilcoxon statistics for the comparison of means, it follows that the differences between the mean responses of Venezuelan professors and those of professors in countries with low GIIs are statistically significant.

The linear regression model does not allow us to explain the responses of the Venezuelan professors from those of the professors in countries with low GIIs with statistical significance, given that the *p*-values of significance of the slopes are less than the 0.05 level of significance (Table 11). Consequently, it can be stated that there is no relationship of dependence, at least linearly expressible, between the two populations of professors with respect to the variables analyzed.

#### 3.2.2. Gender Gap in the Venezuelan Case

The Wilcoxon test for comparison of means did not find significant gender gaps between the female and male Venezuelan professors with respect to their digital competence or assessment of professional aspects (Table 12). However, there was a gender gap in terms of digital stress derived from the pandemic, which was significantly higher among females, whose score in this regard exceeded that of males by 16.74% (Table 12).

Generally, there were no gender gaps in the responses of the Venezuelan professors on digital competence and assessment of professional aspects (Table 12); however, there were gender gaps in this regard within the different areas of knowledge analyzed (Table 13). Specifically, the females express greater digital competence than the males in Health and Social Sciences (with scores 17.80% and 25.90% higher, respectively, than males). In the rest of the areas, it is the males who outperform females in digital competence. This superiority is especially wide in the areas of Sciences (37.14%) and Engineering (11.59%), and more slight in the case of Humanities (2.53%). In any case, the multifactor ANOVA test shows that these gaps are significant (*F* = 26.6502; *p*-value < 0.0001). As for the evaluation of professional aspects, the males outperform the females in the scientific-technical areas: Sciences (with an average difference of 33.50%), Health Sciences (with 12.36%), and Engineering (with 36.48%). In contrast, the female professors in Humanities and Social Sciences value professional aspects less than their male colleagues (with a difference of 2.67% and 47.47%, respectively). 

The multifactor ANOVA test shows that these gaps are significant (*F* = 6.3011, *p*-value < 0.0001). Females have higher levels of pandemic-related digital stress than males in all areas except Health Sciences, in which their mean stress level is intermediate–low and slightly below half that of males (Table 13). In the rest of the areas, the rating given by females to their digital stress exceeds that of males. This difference is notably wide in Engineering (74.46%) and more moderate in Humanities (16.50%), Sciences (17.37%), and Social Sciences (16.32%). These gender gaps in digital stress levels are also statistically significant (*F* = 24.1130, *p*-value < 0.0001). No statistically significant differences by gender are identified in the levels of teaching stress of Venezuelan professors in either private or public universities, according to the statistics of the multifactor ANOVA test (*F* = 2.9410, *p*-value = 0.0867). However, there are differences in digital competence (*F* = 4.4786, *p*-value = 0.0345). Specifically, Venezuelan professors in private universities report higher digital competence than those in public universities (Table 14) and female professors in private universities report higher digital competence than males; in contrast, in public universities, male professors report higher digital competence than females (Table 14). Venezuelan professors at private universities give greater value to the professional aspects involved in their digital teaching activities, but no significant differences by gender are identified on this scale (*F* = 3.1921, *p*-value = 0.0748).

#### 3.2.3. Gender Gap in Low-GII Countries

In contrast to the case in Venezuela, in countries with low GIIs, gender gaps are identified in the three variables analyzed (Table 15). Specifically, the female professors express having greater digital competence than the male professors and also value the professional aspects that influence the digitization of higher education more than the male professors. However, the males report having suffered greater digital stress due to the pandemic because of changes to their teaching, rating their stress about 11% higher than the females did. 

The superiority in digital competence expressed by the females over the males occurs only in the areas of Sciences and Social Sciences, in which the mean ratings of the females are, respectively, 12.68% and 21.52% higher than those of males (Table 16). From the statistics of the multifactor ANOVA test. it follows that this gap is statistically significant (*F* = 24.131, *p*-value < 0.0001). Regarding the assessment of professional aspects, females give higher ratings in all areas of knowledge, and the area of Health Sciences is the one in which the gap with males (of 57.51%) is the greatest. However, in this variable, the multifactor ANOVA test does not reveal statistically significant gaps by knowledge area (*F* = 2.3226, *p*-value = 0.0562). Regarding the incidence of digital stress, it is higher among the males than the females in Humanities and Health Sciences, with mean distances of 42.4% and 82.16%, respectively. In the rest of the areas, the females express greater digital stress than the males, but with very small distances, except in Social Sciences, in which the distance reaches 15.56%. In any case, the differences identified are significant (*F* = 20.7835, *p*-value < 0.0001).

In countries with low GIIs, no significant differences were identified in the behavior of gender gaps between private and public universities with respect to the variables studied (Table 17). Indeed, the multifactor ANOVA test yields *p*-values greater than the level of significance in the assessment of digital competence (*F* = 1.3572, *p*-value = 0.2442), as well as in that of professional aspects (*F* = 0.3574, *p*-value = 0.5503) and in the level of digital stress (*F* = 1.4508, *p*-value = 0.2287). However, there are significant differences that favor professors at private universities between the digital competence ratings of professors at both types of universities (*F* = 242.2332, *p*-value < 0.0001), with mean values of 4.26 out of 5 in private universities and 3.45 out of 5 in public universities.

## 4. Discussion

The COVID-19 pandemic [1] has indeed been a disruptive element in educational activities, especially in universities in Venezuela and in countries with a low level of digitalization, in terms of GIIs [12,26]. Latin American countries [4,5,6] have not been an exception in this regard, despite their levels of digitization [5,28]. The results obtained reveal that the levels of digital competence self-perceived by professors have not moved much due to the pandemic in the countries analyzed, from which it follows that probably not much progress has been made in the process of the digital integration of higher education [10,11,12]. Likewise, the problem of digital stress that studies in the literature have identified is derived from an abrupt digitalization process imposed by circumstances [18,19,20,21,22] and has had a moderate impact among the teachers under study.

The results show that the digital competence of the participating professors is intermediate, as suggested by the strong digital gap and the limitations of technological development in low-GII countries [27,28,30,32] and, especially, in Venezuela [34,35]. In both regions, professors’ levels of digital stress are intermediate (Table 10), which is in line with the results of previous studies conducted in countries with low GIIs [12]. In addition, professors in Venezuela report lower levels of digital competence in teaching than their colleagues in countries with low GIIs, as well as giving a lower value to the professional aspects linked to the incorporation of digital tools into their teaching activities during the pandemic (Table 10). 

In addition, the levels of digital stress due to the pandemic are lower among professors in Venezuela than among those in countries with low GIIs, which could be explained precisely by the lower digital competence of the former, which is associated with a lower implementation of digital technologies in higher education [36]. This is supported by the strong linear dependence observed between stress and digital competence in Venezuelan professors (Table 8). However, the digital competence of professors can only partially explain the levels of digital stress, given that it has also been shown that: (i) the responses of professors in countries with low GIIs do not explain those of professors in Venezuela (Table 11); and (ii) unlike the case of Venezuela, the digital stress of professors in countries with low GIIs is not explained solely by their digital competence (Table 9). 

In the two regions studied (Venezuela and low GII countries), there are gender gaps in the levels of digital stress due to the pandemic, but these are in opposite directions. Specifically, in Venezuela, the females report greater digital stress than the males (Table 12), while in low GII countries, it is the males who report greater digital stress due to the pandemic (Table 15). This shows that the results obtained for countries with low GIIs are in line with those of previous literature [12], while in the case of Venezuela, the opposite is true. Furthermore, in countries with low GIIs, this gap is not associated with gender gaps in terms of digital competence, or the valuation of professional aspects linked to digitalization, contrary to what is reported in the literature for the Latin American and Caribbean regions [39,44].

Regarding the gender gap in digital stress due to the pandemic in different areas of knowledge, the results show that there are two main differences between Venezuela and countries with low GIIs: (i) the direction of the gender gap changes with geographic area in the area of Humanities (among professors in Venezuela, females professors of Humanities have more stress than males, while in countries with low GIIs, the direction of the gap is just the opposite) (Table 13 and Table 16); and (ii) in Venezuela, the direction of the gender gap in digital stress is inverse to that of digital competence in all areas, while in countries with low GIIs, the direction is the same, except in the area of Engineering (Table 13 and Table 16). These facts demonstrate that the gender gap in digital stress due to the pandemic and the digital competence of professors in different areas of knowledge are different in Venezuela and in countries with low GIIs: Bolivia, Honduras, and Guatemala. The specialized literature had already proved the existence of significant differences by professors’ area of knowledge in terms of professors’ digital competence [12,72,73], the digital stress derived from the pandemic [12], the impact of the pandemic on the frequency of use of digital technologies by professors [74], and the assessment of the use of digital tools in the classroom and the capacity of the teaching staff to adapt to them while maintaining a didactic perspective focused on student learning [75,76,77]. However, the description of the reasons that explain this difference in behavior exceeds the purposes of this paper, and they constitute an interesting line of future research. In addition, Health Sciences is the only subject area in which the males present higher digital stress due to their teaching activity after the pandemic in the two geographic areas studied. This result is novel in the literature and constitutes an original contribution of the present investigation. It would be necessary to deepen the descriptive analysis to identify the specific reasons that distinguish the area of Health Sciences from the rest of the areas in the above sense.

It has been shown that male professors in public universities in Venezuela report higher digital competence than their female colleagues, while in Venezuelan private universities it is the females who report higher digital competence (Table 14). In contrast, in countries with low GIIs, female professors rate their digital competence higher than males in both public and private universities (Table 17). This implies that Venezuelan public universities need to make a greater effort to access digital technologies and train their female professors in their usage than private universities. This observation is consistent with works describing the digitization process of higher education in Latin America [80]. As far as it has been possible to explore, the literature does not include works focused on digital competence and pandemic digital stress in the teaching staff of private and public Venezuelan universities, so the results presented here are novel in this regard. Regarding digital stress derived from the pandemic, there are no significant differences between private and public universities in any of the four countries analyzed, although the differences between the genders are wider in private universities, both in Venezuela and in the other countries with low GIIs: Bolivia, Honduras, and Guatemala (Table 14 and Table 17).

Consequently, it can be established that the impact the COVID-19 pandemic has had on the development of digital teacher stress and the gaps observed in that impact are the result of a structural crisis affecting the regions studied; this concept has been established in the preceding literature as having to do with the structural characteristics of societies and educational systems [81]. Indeed, the different incidences of digital teaching stress are related, on the one hand, to the funding that states and universities allocate to the digitization of educational processes, which connects with the development objectives of these states. On the other hand, it is related to certain characteristics strongly rooted in societies, such as gender stereotypes in the professional and, particularly, technological fields.

The main limitation of the study is the lack of homogeneity in the distribution of the participants by gender and university tenure, which could eventually give rise to biases in the results. Likewise, the geographical location of the participants limits the extension of the results. For main lines of future research, the following can be suggested: (i) increase the size of the sample of professors, in order to strengthen the representativeness of the results; (ii) homogenize the sample in terms of the areas of knowledge represented, in order to avoid possible biases that could arise when, as in the present study, the representation of the different areas is not homogeneous; (iii) complement the quantitative analysis with a qualitative study that would help to identify the reasons underlying the identified gender gaps; (iv) deepen the analysis of the situation of the process of technification of Venezuelan higher education and the pandemic digital stress of professors, in order to describe the latent factors in the digital stress of Venezuelan professors, beyond their digital skills.

## 5. Conclusions

Venezuelan university professors report lower digital competence than their colleagues from Latin American countries with low levels of digitalization. The level of digital stress derived from the COVID-19 pandemic is also lower in Venezuelan professors, a phenomenon that may be due to the limitation that the literature attributes to the Venezuelan economy for the integration of digital technologies in higher education.

The digital stress of Venezuelan professors decreases when their digital competence increases and increases when their assessment of the professional aspects linked to the digitization of teaching increases. In contrast, in professors from countries with low levels of digitalization, digital stress derived from the pandemic cannot be explained by appealing to the above factors, but requires additional factors, the identification of which requires further study. Female professors in Venezuela have suffered almost 17% more than male professors in terms of digital stress derived from the pandemic, although the increase rises to almost 75% in Engineering, and up to almost 30% among professors in public universities. On the other hand, in Latin American countries with a low level of digitization, it is the male professors who have suffered the most digital stress, mainly in the areas of Humanities and Health Sciences, but with no significant differences between private and public universities.

From the above results, it follows that, probably, the level of Venezuelan professors’ digital stress in the pandemic is not higher because their universities are experiencing a stagnation in the digitization process. Consequently, it is advisable to increase the funding of Venezuelan universities in the digitization of teaching processes. It is expected that this increase in digitization will be linked to an increase in the digital stress of professors, so that digital integration must be accompanied by: (i) intense training activities for professors to develop digital and techno-pedagogical skills; and (ii) the establishment by universities of well-defined procedures for the use of different digital tools in order to minimize the responsibility of teachers and thus reduce their stress levels. Female professors are the ones who have developed higher levels of stress, which makes it evident that a strong gender gap persists, especially in Venezuela, in terms of women’s access to technologies. This suggests, in turn, two necessary lines of action: (i) in the short term, promoting the use of digital tools among female professors; and (ii) in the long term, carrying out campaigns to incorporate the use of technologies among girls, which will gradually correct the identified gap.

Consequently, it is recommended that universities increase the digital training of professors, especially in terms of developing techno-pedagogical skills. Likewise, it would be convenient for universities to develop closed protocols regarding the use of certain learning platforms to relieve professors of certain kinds of decision making, such as which tools to use for each activities, and to focus on faculty training, in that sense. 

## Figures and Tables

**Table 1:** Countries in Latin America and the Caribbean and their corresponding GIIs.

Country	GII (out of 100)
Chile	36.1
Mexico	34.5
Costa Rica	34.5
Brazil	34.2
Uruguay	32.2
Colombia	31.7
Peru	31.2
Argentina	29.8
Panama	28.0
Paraguay	26.4
Ecuador	25.4
El Salvador	25.0
Guatemala	24.1
Bolivia	23.4
Honduras	22.8

**Table 2:** Questions of the research instrument.

Variable	Number	Questions
Digital competence	1	Rate your digital skills
	2	Rate your ability to adapt to digital learning environments
	3	Rate your capacity for continuous learning
	4	Rate your digital communication skills
	5	Rate your creativity when using digital teaching resources
	6	Rate your knowledge of information management
	7	Rate your network leadership
	8	Rate your ability to orient your didactic actions towards the student in digital environments
	9	Rate your resilience
	10	Rate your ability to teamwork
	11	Rate your strategic vision
Professional aspects	12	Value the support of the university in the process of digitization of teaching
	13	Value the technical equipment of the university
	14	Value the training received in digital matters to face the digitalization process
Digital stress	15	I feel insecure
	16	I feel anxious
	17	I feel that difficulties are piling up
	18	I feel unable to face the new challenges of the digitalization of teaching
	19	I feel irritable
	20	I feel more nervous than usual
	21	I feel anxious about the risk of contagion
	22	I do not feel able to control the situation

**Table 3:** Factors identified by the EFA and factorial weights.

Questions	Factor 1 DigitalCompetence	Factor 2 Professional Aspects	Factor 3 Digital Stress
Rate your digital skills	0.752		
Rate your ability to adapt to digital learning environments	0.623		
Rate your capacity for continuous learning	0.876		
Rate your digital communication skills	0.888		
Rate your creativity when using digital teaching resources	0.809		
Rate your knowledge of information management	0.892		
Rate your network leadership	0.816		
Rate your ability to orient your didactic actions towards the student in digital environments	0.797		
Rate your resilience	0.704		
Rate your ability to teamwork	0.785		
Rate your strategic vision	0.848		
Value the support of the university in the process of digitization of teaching		0.692	
Value the technical equipment of the university		0.820	
Value the training received in digital matters to face the digitalization process		0.802	
I feel insecure			0.782
I feel anxious			0.708
I feel that difficulties are piling up			0.736
I feel unable to face the new challenges of the digitalization of teaching			0.616
I feel irritable			0.694
I feel more nervous than usual			0.860
I feel anxious about the risk of contagion			0.627
I do not feel able to control the situation			0.743

**Table 4:** Cumulative proportion of explained variance of the principal component analysis.

	Digital Competence	Professional Aspects	Digital Stress
Proportion variance	0.095	0.354	0.190
Cumulative variance	0.095	0.449	0.639

**Table 5:** Cronbach’s alphas and CR parameters.

Variable	Cronbach Alpha	CR
Digital competence	0.9587	0.9419
Professional aspects	0.7995	0.7801
Digital stress	0.8880	0.8820

**Table 6:** Descriptive statistics of the responses of the professors in Venezuela.

Variable	Mean (Out of 5)	Standard Deviation (Out of 5)	Coefficient of Variation	Skewness
Digital competence	3.61	0.97	26.96	−0.39
Professional aspects	2.90	1.25	43.18	0.07
Digital stress	2.48	1.26	50.64	0.50

**Table 7:** Lilliefors normality test statistics.

Variable	Lilliefors D	Lilliefors *p*-value
Digital competence	0.2215	<0.0001
Professional aspects	0.1513	<0.0001
Digital stress	0.2214	<0.0001

**Table 8:** Mixed linear regression model statistics of digital stress with respect to digital competence and the valuation of professional aspects in Venezuelan professors.

Variable	Estimate	Std. Error	*t*-Value	$p(>\left|t\right|)$
Digital competence	−0.3277	0.0788	−4.1570	<0.0001
Professional aspects	0.2723	0.0563	4.8330	<0.0001
Independent term	3.3087	0.2825	11.7130	<0.0001

**Table 9:** Mixed linear regression model statistics of digital stress with respect to digital competence and the valuation of professional aspects in professors from countries with low GIIs: Bolivia, Honduras, and Guatemala.

Variable	Estimate	Std. Error	*t*-Value	$p(>\left|t\right|)$
Digital competence	−0.0414	0.0659	−0.6280	0.5304
Professional aspects	−0.1129	0.0553	−2.0430	0.0515
Independent term	3.6231	0.2930	12.3650	<0.0001

**Table 10:** Mean values (out of 5) and statistics of the bilateral Wilcoxon test for the comparison of means between professors from Venezuela and professors from Latin American countries with low GIIs: Bolivia, Honduras, and Guatemala.

Variable	Venezuela	Low GII Countries	Wilcoxon W	Wilcoxon *p*-Value
Digital competence	3.61	3.94	1,247,934	<0.0001
Professional aspects	2.90	3.43	95,100	<0.0001
Digital stress	2.48	2.75	620,340	<0.0001

**Table 11:** Linear regression model to explain the responses of Venezuelan professors based on those of professors in countries with low GIIs (Bolivia, Honduras, and Guatemala) for each of the variables analyzed.

Variable		Estimate	Std. Error	*t*-Value	$p(>\left|t\right|)$
Digital competence	Slope	0.03	0.03	1.06	0.2880
	Independent term	3.85	0.10	35.52	<0.0001
Professional aspects	Slope	0.07	0.04	1.63	0.1040
	Independent term	3.26	0.14	23.65	<0.0001
Digital stress	Slope	0.05	0.03	1.88	0.0604
	Independent term	2.63	0.08	32.64	<0.0001

**Table 12:** Mean values (out of 5) and statistics of the bilateral Wilcoxon test for comparison of means between female and male professors from Venezuela.

Variable	Mean (Females)	Mean (Males)	Wilcoxon W	Wilcoxon *p*-Value
Digital competence	3.58	3.64	254,021	0.4327
Professional aspects	2.92	2.88	18,162	0.7743
Digital stress	2.65	2.27	108,311	<0.0001

**Table 13:** Mean responses (out of 5) of Venezuelan professors differentiated by areas of knowledge and gender.

Variable	Humanities		Sciences		Health		Social Sciences		Engineering	
	F	M	F	M	F	M	F	M	F	M
Digital competence	3.56	3.65	2.45	3.36	3.64	3.09	3.84	3.05	3.97	4.43
Professional aspects	3.08	3.00	2.00	2.67	2.67	3.00	3.20	2.17	2.44	3.33
Digital stress	2.33	2.00	2.50	2.13	1.88	4.13	3.35	2.88	3.21	1.84

**Table 14:** Mean responses (out of 5) of Venezuelan professors differentiated by university tenure and gender.

Variable	Private		Public	
	Females	Males	Females	Males
Digital competence	4.02	3.84	3.49	3.57
Professional aspects	3.67	3.13	2.77	2.79
Digital stress	2.81	2.18	2.61	2.30

**Table 15:** Mean values (out of 5) and statistics of the bilateral Wilcoxon test for comparison of means between female and male professors from countries with low GIIs: Bolivia, Honduras, and Guatemala.

Variable	Mean (Females)	Mean (Males)	Wilcoxon W	Wilcoxon *p*-Value
Digital competence	4.08	3.76	216,568	<0.0001
Professional aspects	3.61	3.19	15,904	0.0027
Digital stress	2.63	2.92	156,448	<0.0001

**Table 16:** Mean responses (out of 5) of professors from countries with low GIIs (Bolivia, Honduras, and Guatemala), differentiated by areas of knowledge and gender.

Variable	Humanities		Sciences		Health		Social Sciences		Engineering	
	F	M	F	M	F	M	F	M	F	M
Digital competence	4.18	4.68	3.91	3.47	3.36	3.50	4.80	3.95	3.55	3.67
Professional aspects	3.89	3.67	3.44	3.27	3.67	2.33	4.00	3.67	3.17	3.00
Digital stress	2.50	3.56	2.69	2.65	2.13	3.88	2.60	2.25	2.78	2.75

**Table 17:** Mean responses (out of 5) of professors from countries with low GIIs (Bolivia, Honduras, and Guatemala), differentiated by university tenure and gender.

Variable	Private		Public	
	Females	Males	Females	Males
Digital competence	4.27	4.24	3.54	3.40
Professional aspects	3.69	3.28	3.40	3.13
Digital stress	2.65	3.08	2.55	2.80

## Data Availability

The data are not publicly available because they are part of a larger project involving more researchers. If you have any questions, please ask the contact author.

## References

[1] Pradhan A., Prabhu S., Chadaga K., Sengupta S., Nath G. (2022). Supervised learning models for the preliminary detection of COVID-19 in patients using demographic and epidemiological parameters. Information.

[2] Alonso-García M., Garrido-Letrán T.M., Sánchez-Alzola A. (2021). Impact of COVID-19 on educational sustainability. Initial perceptions of the university community of the University of Cádiz. Sustainability.

[3] Haleem A., Javaid M., Vaishya R. (2020). Effects of COVID-19 pandemic in daily life. Curr. Med. Res. Pract..

[4] Sahu P. (2020). Closure of universities due to coronavirus disease 2019 (COVID-19): Impact on education and mental health of students and academic staff. Cureus.

[5] Verma G., Campbell T., Melville W., Park B.-Y. (2020). Science teacher education in the times of the COVID-19 pandemic. J. Sci. Teach. Educ..

[6] Paudel P. (2021). Online education: Benefits, challenges and strategies during and after COVID-19 in higher education. Int. J. Stud. Educ..

[7] MacIntyre P.D., Gregersen T., Mercer S. (2020). Language professors’ coping strategies during the COVID-19 conversion to online teaching: Correlations with stress, wellbeing and negative emotions. System.

[8] Blume C. (2020). German professors’ digital habitus and their pandemic pedagogy. Postdigit. Sci. Educ..

[9] Ferdig R.E., Baumgartner E., Hartshorne R., Kaplan-Rakowski R., Mouza C. (2020). Teaching, Technology, and Teacher Education during the COVID-19 Pandemic: Stories from the Field.

[10] Vlasova E.Z., Barakhsanova E.A., Goncharova S.V., Ilina T.S., Aksyutin P.A. (2020). Teacher education in higher education systems during pandemic and the synergy of digital technology. Propósitos Y Represent..

[11] Khan M.A., Omrane A., Omrane A., Kassmi K., Akram M.W., Khanna A., Mostafiz M.I. (2020). The era of a digitalized world. Sustainable Entrepreneurship, Renewable Energy-Based Projects, and Digitalization.

[12] Antón-Sancho Á., Vergara D., Fernández-Arias P. (2022). Influence of country digitization level on digital pandemic stress. Behav. Sci..

[13] Pokhrel S., Chhetri R. (2021). A literature review on impact of COVID-19 pandemic on teaching and learning. High. Educ. Future.

[14] Tadesse S., Muluye W. (2020). The impact of COVID-19 pandemic on education system in developing countries: A review. Open J. Soc. Sci..

[15] Antón-Sancho Á., Vergara D., Lamas-Álvarez V.E., Fernández-Arias P. (2021). Digital content creation tools: American university professors’ perception. Appl. Sci..

[16] Eri R., Gudimetla P., Star S., Rowlands J., Girgla A., To L., Li F., Sochea N., Bindal U. (2021). Digital resilience in higher education in response to COVID-19 pandemic: Student perceptions from Asia and Australia. J. Univ. Teach. Learn. Pract..

[17] Toader T., Safta M., Titirișcă C., Firtescu B. (2021). Effects of digitalisation on higher education in a sustainable development framework—Online learning challenges during the COVID-19 pandemic. Sustainability.

[18] Robinson L., Schulz J., Wiborg Ø.N., Johnston E. (2021). The COVID connection: Pandemic anxiety, COVID-19 comprehension, and digital confidence. Am. Behav. Sci..

[19] Lystras M.D., Serban A.C., Torres-Ruiz M.J., Ntanos S., Srirete A. (2022). Translating knowledge into innovation capability: An exploratory study investigating the perceptions on distance learning in higher education during the COVID-19 pandemic—the case of Mexico. J. Innov. Knowl..

[20] Händel M., Stephan M., Gläser-Zikuda M., Kopp B., Bedenlier S., Ziegler A. (2022). Digital readiness and its effects on higher education students’ socio-emotional perceptions in the context of the COVID-19 pandemic. J. Res. Technol. Educ..

[21] Okoye K., Rodriguez-Tort J.A., Escamilla J., Hosseini S. (2021). Technology-mediated teaching and learning process: A conceptual study of educators’ response amidst the COVID-19 pandemic. Educ. Inf. Technol..

[22] Martzoukou K., Fulton C., Kostagiolas P., Lavranos C. (2020). A study of higher education students’ self-perceived digital competences for learning and everyday life online participation. J. Doc..

[23] Panisoara I.O., Lazar I., Panisoara G., Chirca R., Ursu A.S. (2020). Motivation and continuance intention towards online instruction among professors during the COVID-19 pandemic: The mediating effect of burnout and technostress. Int. J. Environ. Res. Public Health.

[24] Vergara-Rodríguez D., Antón-Sancho Á., Fernández-Arias P. (2022). Variables influencing professors’ adaptation to digital learning environments during the COVID-19 pandemic. Int. J. Environ. Res. Public Health.

[25] Essel H.B., Vlachopoulos D., Tachie-Menson A., Johnson E.E., Ebeheakey A.K. (2021). Technology-induced stress, sociodemographic factors, and association with academic achievement and productivity in Ghanaian higher education during the COVID-19 pandemic. Information.

[26] WIPO (2021). The Global Innovation Index 2021: Tracking Innovation through the COVID-19 Crisis.

[27] Valdés K.N., Alpera S.Q., Cerdá-Suárez L.M. (2021). An institutional perspective for evaluating digital transformation in higher education: Insights from the Chilean case. Sustainability.

[28] Katz R.L., Koutroumpis P., Callorda F. (2013). The Latin American path towards digitization. Info.

[29] Cerdá-Suárez L.M., Núñez-Valdés K., Quirós y Alpera S. (2021). A systemic perspective for understanding digital transformation in higher education: Overview and subregional context in Latin America as evidence. Sustainability.

[30] Basilotta-Gómez-Pablos V., Matarranz M., Casado-Aranda L.-A., Otto A. (2022). Professors’ digital competencies in higher education: A systematic literature review. Int. J. Educ. Technol. High. Educ..

[31] Antón-Sancho Á., Vergara D., Fernández-Arias P. (2021). Self-assessment of soft skills of university professors from countries with a low level of digital competence. Electronics.

[32] Cabero-Almenara J., Llorente-Cejudo C. (2020). COVID-19: Radical transformation of digitization in university institutions. Campus Virtuales.

[33] The Global Economy. https://www.theglobaleconomy.com/Venezuela/GII_Index/.

[34] Prince-Machado M.S., Tenorio-Sepúlveda G.C., Ramirez-Montoya M.S. (2016). Educational innovation and digital competencies: The case of OER in a private Venezuelan university. Int. J. Educ. Technol. High. Educ..

[35] Parga-García R.A. (2020). Distance education in a time of pandemic: Viability in the current context of education in Venezuela. Rev. De Cienc. De La Educ..

[36] Crawford J., Butler-Henderson K., Rudolph J., Malkawi B., Glowatz M., Burton R., Magni P., Lam S. (2020). COVID-19: 20 countries’ higher education intra-period digital pedagogy responses. J. Appl. Learn. Teach..

[37] Abuelafia E., Saboin J.L. (2020). A Look to the Future for Venezuela.

[38] Abuelafia E., Saboin J.L. (2021). Los Desafíos Para la Recuperación de Venezuela y el Impacto del COVID-19.

[39] Basantes-Andrade A., Cabezas-González M., Casillas-Martín S. (2020). Digital competences relationship between gender and generation of university professors. Int. J. Adv. Sci. Eng. Inf. Technol..

[40] Contreras-Ortiz S., Villa-Ramírez J.L., Osorio-Delvalle C., Ojeda-Caicedo V. Participation of women in STEM higher education programs in Latin America: The issue of inequality. Proceedings of the 18th LACCEI International Multi-Conference for Engineering Education, and Technology, Paper presented at LACCEI: Virtual Edition.

[41] Hilbert M. (2011). Digital gender divide or technologically empowered women in developing countries? A typical case of lies, damned lies, and statistics. Women’s Stud. Int. Forum.

[42] García-Holgado A., Camacho-Díaz A., García-Peñalvo F.J. Engaging women into STEM in Latin America: W-STEM project. Proceedings of the Seventh International Conference on Technological Ecosystems for Enhancing Multiculturality, Paper presented at TEEM’19, Association for Computing Machinery.

[43] Ramírez-Lozano J.P., Bridshaw-Araya L.C., Brito-Ochoa M.P. (2022). Latin American female academic perceptions about the COVID pandemic’s impact on gender equity and within-country inequality. Manag. Res..

[44] Portillo J., Garay U., Tejada E., Bilbao N. (2020). Self-perception of the digital competence of educators during the COVID-19 pandemic: A cross-analysis of different educational stages. Sustainability.

[45] Peters S., Jornitz S., Parreira do Amaral M. (2021). The education system of Venezuela. The Education Systems of the Americas.

[46] Bienertova-Vasku J., Lenart P., Scheringer M. (2020). Eustress and distress: Neither good nor bad, but rather the same?. BioEssays.

[47] Fernández-Suárez I., García-González M.A., Torrano F., García-González G. (2021). Study of the prevalence of burnout in university professors in the period 2005–2020. Educ. Res. Int..

[48] Kwon K.-A., Ford T.G., Tsotsoros J., Randall K., Malek-Lasater A., Kim S.G. (2022). Challenges in working conditions and well-being of early childhood professors by teaching modality during the COVID-19 pandemic. Int. J. Environ. Res. Public Health.

[49] Maslach C., Vandenberghe R., Huberman A. (1999). Progress in understanding teacher burnout. Understanding and Preventing Teacher Burnout: A Sourcebook of International Research and Practice.

[50] Besser A., Lotem S., Zeigler-Hill V. (2022). Psychological stress and vocal symptoms among university professors in Israel: Implications of the shift to online synchronous teaching during the COVID-19 pandemic. J. Voice.

[51] Swigonski N.L., James B., Wynns W., Casavan K. (2021). Physical, mental, and financial stress impacts of COVID-19 on early childhood educators. Early Child. Educ. J..

[52] Aperribai L., Cortabarria L., Aguirre T., Verche E., Borges A. (2020). Teacher’s Physical Activity and Mental Health During Lockdown Due to the COVID-2019 Pandemic. Front. Psychol..

[53] Alves R., Lopes T., Precioso J. (2021). Professors’ well-being in times of COVID-19 pandemic: Factors that explain professional well-being. IJERI: Int. J. Educ. Res. Innov..

[54] Kim L.E., Asbury K. (2020). Like a rug had been pulled from under you’: The impact of COVID-19 on teachers in England during the first six weeks of the UK lockdown. Br. J. Educ. Psychol..

[55] Burić I., Kim L.E. (2020). Teacher self-efficacy, instructional quality, and student motivational beliefs: An analysis using multilevel structural equation modeling. Learn. Instr..

[56] Kwon K.-A., Jeon S., Jeon L., Castle S. (2019). The role of teachers’ depressive symptoms in classroom quality and child developmental outcomes in Early Head Start programs. Learn. Individ. Differ..

[57] Jeon H.-J., Kwon K.-A., Walsh B., Burnham M.M., Choi Y.-J. (2019). Relations of early childhood education teachers’ depressive symptoms, job-related stress, and professional motivation to beliefs about children and teaching practices. Early Educ. Dev..

[58] Collie R.J. (2021). COVID-19 and teachers’ somatic burden, stress, and emotional exhaustion: Examining the role of principal leadership and workplace buoyancy. AERA Open.

[59] Sokal L., Trudel L.E., Babb J. (2020). Canadian teachers’ attitudes toward change, efficacy, and burnout during the COVID-19 pandemic. Int. J. Educ. Res. Open.

[60] Sokal L.J., Trudel L.G.E., Babb J.C. (2020). Supporting teachers in times of change: The job demands- resources model and teacher burnout during the COVID-19 pandemic. Int. J. Contemp. Educ..

[61] Puertas-Molero P., Zurita-Ortega F., Chacón-Cuberos R., Martínez-Martínez A., Castro-Sánchez M., González-Valero G. (2018). An explanatory model of emotional intelligence and its association with stress, burnout syndrome, and non-verbal communication in the university teachers. J. Clin. Med..

[62] Chan D.W. (2006). Emotional intelligence and components of burnout among Chinese secondary school teachers in Hong Kong. Teach. Teach. Educ..

[63] Zysberg L., Orenshtein C., Gimmon E., Robinson R. (2017). Emotional intelligence, personality, stress, and burnout among educators. Int. J. Stress Manag..

[64] Madaliyeva Z., Mynbayeva A., Sadvakassova Z., Zholdassova M. (2015). Correction of Burnout in Teachers. Procedia Soc. Behav. Sci..

[65] Vesely A.K., Saklofske D.H., Nordstokke D.W. (2014). EI training and pre-service teacher wellbeing. Personal. Individ. Differ..

[66] Pena-Garrido M., Extremera-Pacheco N. (2011). Perceived emotional intelligence in primary school teachers and its relationship with levels of burnout and engagement. Rev. De Educ..

[67] Ju C., Lan J., Li Y., Feng W., You X. (2015). The mediating role of workplace social support on the relationship between trait emotional intelligence and teacher burnout. Teach. Teach. Educ..

[68] Rogowska A.M., Meres H. (2022). The mediating role of job satisfaction in the relationship between emotional intelligence and life satisfaction among teachers during the COVID-19 pandemic. Eur. J. Investig. Health Psychol. Educ..

[69] Brackett M.A., Palomera R., Mojsa-Kaja J., Reyes M.R., Salovey P. (2010). Emotion-regulation ability, burnout, and job satisfaction among British secondary-school teachers. Psychol. Sch..

[70] Feuerhahn N., Stamov-Roßnagel C., Wolfram M., Bellingrath S., Kudielka B.M. (2013). Emotional exhaustion and cognitive performance in apparently healthy teachers: A longitudinal multi-source study. Stress Health.

[71] Puertas-Molero P., Zurita-Ortega F., Ubago-Jiménez J.L., González Valero G. (2019). Influence of emotional intelligence and burnout syndrome on teachers well-being: A systematic review. Soc. Sci..

[72] Fernández-Arias P., Antón-Sancho Á., Vergara D., Barrientos A. (2021). Soft skills of American university teachers: Self-concept. Sustainability.

[73] Antón-Sancho Á., Vergara D., Fernández-Arias P., Ariza-Echeverri E.A. (2022). Didactic use of virtual reality in Colombian universities: Professors’ perspective. Multimodal Technol. Interact..

[74] Antón-Sancho Á., Sánchez-Calvo M. (2022). Influence of knowledge area on the use of digital tools during the COVID-19 pandemic among Latin American professors. Educ. Sci..

[75] Antón-Sancho Á., Fernández-Arias P., Vergara D. (2022). Assessment of virtual reality among university professors: Influence of the digital generation. Computers.

[76] Vergara D., Antón-Sancho A., Dávila L.P., Fernández-Arias P. (2022). Virtual reality as a didactic resource from the perspective of engineering teachers. Comput. Appl. Eng. Educ..

[77] Vergara D., Antón-Sancho A., Fernández-Arias P. (2022). Player profiles for game-based applications in engineering education. Comput. Appl. Eng. Educ..

[78] Zhao Y. (2020). COVID-19 as a catalyst for educational change. Prospects.

[79] Bennett L. (2014). Putting in more: Emotional work in adopting online tools in teaching and learning practices. Teach. High. Educ..

[80] Rama C. (2014). University virtualisation in Latin America. Int. J. Educ. Technol. High. Educ..

[81] Petousi V., Sifaki E. (2020). Contextualizing harm in the framework of research misconduct. Findings from a discourse analysis of scientific publications. Int. J. Sustain. Dev..

[82] UNESCO Institute for Statistics (2012). International Standard Classification of Education ISCED 2011.

